# Microstructure and mineral composition of dystrophic calcification associated with the idiopathic inflammatory myopathies

**DOI:** 10.1186/ar2841

**Published:** 2009-10-26

**Authors:** Naomi Eidelman, Alan Boyde, Andrew J Bushby, Peter GT Howell, Jirun Sun, Dale E Newbury, Frederick W Miller, Pamela G Robey, Lisa G Rider

**Affiliations:** 1 Paffenbarger Research Center, American Dental Association Foundation, National Institute of Standards and Technology, 100 Bureau Drive, Stop 8546, Gaithersburg, MD 20899, USA; 2 Biophysics OGD, Dental Institute, Queen Mary University of London, New Road, London E1 1BB, UK; 3 Department of Materials, Queen Mary University of London, Mile End Road, London E1 4NS, UK; 4 Prosthetic Dentistry Unit, UCL Eastman Dental Institute, 256 Gray's Inn Road, London WC1X 8LD, UK; 5 Polymers Division, National Institute of Standards and Technology, 100 Bureau Drive, Stop 8543, Gaithersburg, MD 20899, USA; 6 Surface and Microanalysis Science Division, National Institute of Standards and Technology, 100 Bureau Drive, Stop 8371, Gaithersburg, MD 20899, USA; 7 Environmental Autoimmunity Group, Office of Clinical Research, 10 Center Drive, MSC 1301, National Institute of Environmental Health Sciences, National Institutes of Health, Bethesda, MD 20892, USA; 8 National Institute of Dental and Craniofacial Research, National Institutes of Health, 30 Convent Drive, Bethesda, MD 20892, USA; 9 Current address: Paffenbarger Research Center, American Dental Association Foundation, National Institute of Standards and Technology, 100 Bureau Drive, Stop 8546, Gaithersburg, MD 20899, USA

## Abstract

**Introduction:**

Calcified deposits (CDs) in skin and muscles are common in juvenile dermatomyositis (DM), and less frequent in adult DM. Limited information exists about the microstructure and composition of these deposits, and no information is available on their elemental composition and contents, mineral density (MD) and stiffness. We determined the microstructure, chemical composition, MD and stiffness of CDs obtained from DM patients.

**Methods:**

Surgically-removed calcinosis specimens were analyzed with fourier transform infrared microspectroscopy in reflectance mode (FTIR-RM) to map their spatial distribution and composition, and with scanning electron microscopy/silicon drift detector energy dispersive X-ray spectrometry (SEM/SDD-EDS) to obtain elemental maps. X-ray diffraction (XRD) identified their mineral structure, X-ray micro-computed tomography (μCT) mapped their internal structure and 3D distribution, quantitative backscattered electron (qBSE) imaging assessed their morphology and MD, nanoindentation measured their stiffness, and polarized light microscopy (PLM) evaluated the organic matrix composition.

**Results:**

Some specimens were composed of continuous carbonate apatite containing small amounts of proteins with a mineral to protein ratio much higher than in bone, and other specimens contained scattered agglomerates of various sizes with similar composition (FTIR-RM). Continuous or fragmented mineralization was present across the entire specimens (μCT). The apatite was much more crystallized than bone and dentin, and closer to enamel (XRD) and its calcium/phophorous ratios were close to stoichiometric hydroxyapatite (SEM/SDD-EDS). The deposits also contained magnesium and sodium (SEM/SDD-EDS). The MD (qBSE) was closer to enamel than bone and dentin, as was the stiffness (nanoindentation) in the larger dense patches. Large mineralized areas were typically devoid of collagen; however, collagen was noted in some regions within the mineral or margins (PLM). qBSE, FTIR-RM and SEM/SDD-EDS maps suggest that the mineral is deposited first in a fragmented pattern followed by a wave of mineralization that incorporates these particles. Calcinosis masses with shorter duration appeared to have islands of mineralization, whereas longstanding deposits were solidly mineralized.

**Conclusions:**

The properties of the mineral present in the calcinosis masses are closest to that of enamel, while clearly differing from bone. Calcium and phosphate, normally present in affected tissues, may have precipitated as carbonate apatite due to local loss of mineralization inhibitors.

## Introduction

Approximately 30% of patients with juvenile dermatomyositis (JDM) develop dystrophic calcification, which is associated with increased functional disability and a chronic illness course [1-3]. Calcinosis has been reported, but less frequently in adult patients [4]. These calcified deposits often develop in sites of microtrauma, including the joint extensor surfaces, digits and extremities, although they may occur anywhere [1]. Several subtypes of calcinosis are recognized, including superficial nodules, tumorous deposits, fascial planar lesions and exoskeleton [5].

Very little is known about the biology of dystrophic calcification in dermatomyositis. Calcinosis is associated with prolonged disease activity in patients with JDM [1], a chronic course of illness [1], TNFα-308A allele (a pro-inflammatory promoter polymorphism) and increased production of TNFα [6], and with IL-1 cytokine polymorphisms [7]. Osteopontin, osteonectin, and bone sialoprotein have been identified in protein extracts from JDM patients [8]. Limited information is available about the microstructure and composition of the calcified deposits in DM specimens and most of the studies were case reports or included small number of patients [8-12]. There are no reports on their mineral density (MD), stiffness and elemental composition mapping.

In order to better characterize the spatial composition, structure, MD, stiffness and distribution of the calcified deposits in surgically removed calcinosis specimens from myositis patients, we have applied: Fourier Transform Infrared microspectroscopy in reflectance mode (FTIR-RM) to map the spatial mineral and organic matrix composition and the distribution of the calcified deposits in the whole cross sections of specimens and at various depths; scanning electron microscopy with silicon drift detector energy dispersive X-ray spectrometry (SEM/SDD-EDS) to acquire maps of the chemical elements; X-ray diffraction (XRD) and polarized light microscopy (PLM) to further characterize the chemical structure of the mineral and the organic matrix composition respectively; quantitative backscattered electron (qBSE) imaging to determine the MD and detailed morphology; nanoindentation to study the mineral stiffness; and X-ray micro-computed tomography (μCT) to obtain the 3D internal structure and distribution of the deposits. This is the first application of FTIR-RM and SEM/SDD-EDS mapping, qBSE imaging and nanoindentation to analyze the composition, microstructure, MD and mechanical strength of DM deposits, and the largest integrated multi-methods, multi specimens study. Studying these calcified deposits using multiple techniques on multiple specimens is the only way to fully understand the mechanism of their formation, and might provide insight into therapeutic intervention for their prevention.

## Materials and methods

Solid calcinosis deposits were studied after informed consent was obtained from patients and, in the case of minors, their parents, in a National Institutes of Health institutional review board approved study. The calcified deposits masses were surgically removed from four patients with JDM, two with adult DM, and two with juvenile polymyositis (JPM; Table 1), with probable or definite myositis according to the Bohan and Peter criteria [13]. Samples from one of the adult DM patients were received post-mortem. Patients and/or their referring physicians provided an estimated duration of the lesions and the treatments. The specimens were fixed in 4% phosphate buffered formaldehyde for 24 to 48 hours, then either stored in 70% ethanol (for FTIR-RM, SEM/SDD-EDS, μCT, XRD, qBSE, nanoindentation) or partially decalcified in 4% EDTA in phosphate buffer (pH 7.0) for PLM.

**Table 1:** Clinical characteristics of myositis patients with calcinosis samples and the methods used to characterize the specimens

*Sample*	*Study performed*	*Location*	*Diagnosis*	*Age at surgical removal*	*Race*	*Gender*	*Duration of myositis*	*Estimated calcinosis duration *	*Medications at time of surgical removal*
Calc1	FTIR-RM qBSE SEM/SDD-EDS PLM	Elbow	JDM	15.5 years	Hispanic	Male	5.8 years	2-3 months	Methylprednisolone, cyclosporine, azathioprine, alendronate, ibuprofen
Calc2	FTIR-RM qBSE SEM/SDD-EDS PLM, μCT	Toe	JPM	5.4 years	Black	Male	2 years	12 months	Prednisone, cyclosporine, rofecoxib, calcium
Calc3	qBSE PLM	Biceps	JDM	11.9 years	Caucasian	Female	4.5 years	9 months	Prednisone, methotrexate, hydroxychloroquine, probenecid
Calc4	qBSE PLM	Hamstring	JPM	16.8 years	Caucasian	Female	4.9 years	> 3 years	Alendronate, celecoxib, diltiazem
Calc6	qBSE PLM	Right buttock	DM	56 years	Korean	Female	4.9 years	6 months	Prednisone
Calc7 *	FTIR-RM XRD, μCT PLM	Elbow and finger	DM	50 years	Caucasian	Female	12 years	Unknown	Prednisone, methotrexate, alendronate, valdecoxib
Calc8	FTIR-RM XRD, μCT PLM	Shoulder	JDM	49 years	Caucasian	Female	30 years	27 years	Nifedipine
Calc10	FTIR-RM PLM	Thigh	JDM	12 years	Caucasian	Female	4 years	3 months	Prednisone, methotrexate, myocophenolate mofetil, alendronate, colchicine, folic acid

Specimens Calc5 and Calc9 were not included in this study. *post-mortem sample DM = dermatomyositis; FTIR-RM = Fourier Transform Infrared microspectroscopy in reflectance mode; JDM = juvenile dermatomyositis; JPM = juvenile polymyositis; PLM = polarized light microscopy; qBSE = quantitative backscattered electron; SEM/SDD-EDS = scanning electron microscopy/silicon drift detector energy dispersive X-ray spectrometry; μCT = X-ray micro-computed tomography; XRD = X-ray diffraction.

Specimens Calc7, Calc8 and Calc10 were first photographed, then freeze-dried and re-photographed. The whitish cut cross section of Calc7 became brittle after lyophilization, enabling us to scrape several bulging white particles for powder XRD analysis. The freeze-dried specimens were embedded in acrylic embedding medium (Ultra-Mount™, Buehler, Lake Bluff, IL, USA) with their largest face down as parallel as possible to the flat bottom of a mold. After curing at room temperature for 24 hours, the cross sections were exposed by dry polishing with abrasive papers sequentially to 4000 grit. The final polished faces were photographed, mapped with FTIR-RM and then underwent XRD and μCT. Blocks from Calc1 and Calc2 were prepared and measured first with qBSE as described below and subsequently mapped with FTIR-RM and SEM/SDD-EDS without any additional polishing.

### FTIR microspectroscopy

FTIR-RM analyses were performed on the cross sections of Calc1, Calc2, Calc7, Calc8 and Calc10 using a Nic-Plan IR microscope interfaced with a Nicolet Magna-IR 550 FTIR spectrophotometer (Nicolet Instrumentations Inc., Madison, WI, USA) operated in reflectance mode, as previously described [14]. FTIR-RM maps [14] were obtained from the surface of the whole polished embedded cross section of the masses in the 650 cm^-1 ^to 4000 cm^-1 ^region with 8 cm^-1 ^resolution, 32 scans per spectrum and beam spot size of 200 × 200 μm to 140 × 140 μm. Higher spatial resolution (80 × 80 μm to 40 × 40 μm) was used to map regions with the highest calcified deposits density. The reflectance spectra were proportioned against background of an aluminum (Al) mirror and transformed to absorbance spectra using the Kramers-Kronig algorithm [15]. The FTIR-RM maps were processed as mineral and proteins maps (area under the ν_3 _PO_4_and under the amides peaks, respectively), and displayed as color contour maps along with the visual map that was obtained with the same FTIR microscope lens. Spectra extracted from calcified regions in the maps were compared with bone, dentin and enamel spectra and to spectra obtained from pressed pellets prepared from synthetic 'physiological' apatite and highly crystallized hydroxyapatite for identification and characterization of the mineral phase. The synthetic apatite, prepared under physiological conditions (37°C, pH 7.4), was donated by Dr ED Eanes, and the fine powder of highly crystallized hydroxyapatite [16] was donated by Mr BO Fowler (both formerly with the National Institute of Dental and Craniofacial Research (NIDCR), National Institutes of Health (NIH)). The peaks of the baseline corrected spectra (800 cm^-1 ^to 1800 cm^-1^) obtained from calcinosis, dentin and bone specimens, were resolved by PeakFit™ (Jandel Corporation, San Rafael, CA, USA), using the second derivative method and the integrated areas of the ν_3 _PO_4 _(980 cm^-1 ^to 1200 cm^-1^) and of amide I (1595 cm^-1 ^to 1720 cm^-1^) bands were calculated. The mineral-to-proteins ratios (PO_4_/amide I) and the amounts of proteins present in the calcified regions relative to bone (Calc/Bone) were determined.

In order to check if additional or different deposits would be present inside the bulk or closer to the edges of the specimens, both top and bottom of the embedded Calc10, and the top and the cut cross section of Calc7 were polished and mapped with FTIR-RM. An additional layer from the top cross section of Calc10 was removed by re-polishing after the first plane was mapped, and its cross section was re-mapped with FTIR-RM.

### Scanning Electron Microscopy with Silicon drift detector energy dispersive X-ray spectrometry

SEM/SDD-EDS performed in the scanning electron microscope (SEM) was carried out using the Bruker QUAD SDD (a cluster of four 10 mm^2 ^SDDs; Berlin, Germany) on the same polished surfaces of specimens Calc1 and Calc2 that were mapped also with FTIR-RM and qBSE, to spatially map the relative concentrations of the chemical elements that are present in these deposits. The SDD, which has a highly efficient charge collection mechanism, permits a factor of 10 to 40 faster pulse processing for the same resolution than Si(Li)-EDS, and modest cooling requirements (250°K). An SEM equipped with a thermal field-emission gun can produce high spatial resolution probes that can carry 10 to 100 times greater beam current, providing high spatial resolution and high X-ray generation rates. The lateral resolution depends on the beam energy and composition, but for the 15 keV energy that was used in this study that was performed on apatite, it was about 1 μm. Lispix, the advanced image processing software developed at NIST (Gaithersburg, MD, USA) by David Bright [17], was used for data analysis. The 'maximum pixel' derived spectrum tool in Lispix can detect an unanticipated element that appears at a single pixel in a huge map and localize it spatially [18]. The surfaces of the specimens that were coated earlier with carbon for the qBSE measurements were coated with additional carbon using high purity carbon, for better contrast. SEM-BSE images and SEM/SDD-EDS calcium (Ca), phosphorous (P), carbon (C), oxygen (O), magnesium (Mg) and sodium (Na) maps were obtained. The relative concentrations of all detected elements (Ca, P, C, O, Mg and Na and the trace elements: chlorine (Cl), Al and sulfur (S) were determined, using the summation (SUM) spectra as defined by the Ca-mask. An X-ray spectrum representing each entire Calc structure was created by first using the Ca map of the structure to define a mask of selected pixels and then summing the spectra from these pixels to create a 'SUM' spectrum. This SUM spectrum was then analyzed quantitatively against standards measured under identical conditions (fluoroapatite for Ca and P; albite for Na, MgO for Mg; FeS_2 _for S; Al metal for Al; KCl for Cl; oxygen was calculated from the cation analyses by assumed stoichiometry) with interelement matrix corrections determined with the NIST electron-excited X-ray microanalysis DTSA II software [19]. The detection limit for F is 0.05%. The limit of detection is based upon the counting statistics of the X-ray continuum background at the energy of the characteristic peak of the element of interest. The standard deviation (SD) is estimated from the counts integrated across the range of channels that define each X-ray peak, considering the contributions to the error budget from both the unknown and standards.

### X-ray diffraction

XRD patterns of particles collected from Calc7 and powders of bone, dentin, enamel and both the apatites used for the FTIR-RM pellets were recorded with CuKα radiation (λ = 0.154 nm) using a Rigaku 2200 D-Max X-ray diffractometer (Rigaku/USA Inc., Danvers, MA, USA) operating at 40 kV and 40 mA at 4° to 65° 2θ range with intervals of 0.010° 2θ. The same divergence and anti-scatter slits (1°) and receiving slit (0.6 mm) were used for all samples. The embedded and polished cross sections of Calc7 and Calc8 underwent XRD after being mapped with FTIR-RM. Porous regions that were mixed with embedding material were covered with Al foil to eliminate the contribution of the embedding material to the XRD pattern of the calcified region. The full width at one half the maximum height (FWHM) above background values of the well resolved 002 diffraction peak (25.8°) of the apatite in Calc7 and Calc8 as well as these of bone, dentin, enamel, physiological and highly crystalline apatites were determined with the Jade 6.1 software (Materials Data, Inc., Livermore, CA, USA) using the Pseudo Voigt function to model the peak shape. As FWHM correlates inversely with crystal size and lattice perfection [20], its reciprocal values (1/FWHM) for the 002 peak and the relative values to these obtained from bone, were used as a comparative quantitative measure of the crystallinity of the mineral present in the calcinosis specimens.

### X-ray microcomputed tomography

The embedded and polished Calc2, Calc7 and Calc8 specimens (2 mm, 3.4 mm and 3.4 mm thick, respectively) that were analyzed with FTIR-RM and qBSE (Calc2) were imaged with an X-ray μCT scanner (Scanco Medical μCT40, Bassersdorf, Switzerland) to acquire 2D and 3D images of the calcified deposits in order to map the internal structure of the specimens. The micro-focus X-ray source was set at 75 kVp and 114 μA and the specimens were scanned at 18 μm line resolution with an integration time of 300 seconds. The specimens were placed horizontally with the polished surfaces upright and were fixed in the μCT sample holder. A series of 2D images were collected and reconstructed into 3D images using the manufacturer's complete imaging and evaluation software and ImageJ image analysis software (version 1.39, NIH, Bethesda, MD, USA).

### Quantitative backscattered electron imaging

Five specimens (Calc1, Calc2, Calc3, Calc4 and Calc6) were dehydrated in ethanol and embedded in poly-methyl methacrylate (PMMA). The resulting blocks were halved and trimmed with a water cooled diamond saw, the flat surfaces polished to 0.25 μm diamond finish, dried and coated with carbon by evaporation. They were imaged using an automated digital SEM (Zeiss DSM 962, Cambridge, UK) operated at 20 kV. Brominated and iodinated dimethacrylates were used as standards against which to assess the qBSE signal, as previously described for studies of urinary stones [21], bone [22-24], calcified cartilage [25], dentin [26], and enamel [27,28]. Preliminary experiments showed that the electron backscattering coefficient for the denser regions of the calcinosis samples lay above those for bone and calcified cartilage and dentin, but below those for dental enamel. We therefore chose to use the mono-brominated and tetra-brominated dimethacrylate resin standards. The gray scale values were then adjusted to cover this range (0 to 255) [24,29,30]. Entire block faces were montaged by scanning multiple adjoining fields.

### Nanoindentation

The MD determined by qBSE imaging was correlated with nanoindentation elastic modulus (E GPa) in arrays of 15 μm spaced sites in selected areas of Calc3 and Calc4. The PMMA blocks were fastened to steel mounts using cyanoacrylate adhesive. Regions of interest were tested using a UMIS 2000 nanoindentation system (CSIRO, Sydney, Australia) using a 5 μm radius spherical geometry diamond indenter tip to a maximum load of 15 mN. The tip shape and frame stiffness were calibrated using a multiple reference material method [31]. Each indentation test consisted of 40 load increments, unloading to 75% of each load between increments where the *plane strain *elastic indentation modulus (*E*) was calculated as a function of contact depth for each load/partial-unload data pair [31,32], and a mean value of modulus was derived for each indentation site from the 20 deepest increments. Arrays of indents spanned poorly to well mineralized soft tissue.

### Matching sites of qBSE and nanoindentation measurements

The indent array sites were chosen by location in the original lower resolution qBSE montage images. After nanoindentation, each field was rescanned twice using BSE-SEM at high resolution (nominal 150 × magnification and 0.29 μm pixel size, 594 μm and 2048 pixels wide), 20 kV accelerating voltage, 0.5 nA probe current, 17 mm working distance, 11 mm detector to sample distance, first in compositional ('A+B') mode by summing the output of all four BSE detector quadrants and then in topographic ('A-B') mode using the difference between opposing detector segment pairs to permit the exact location of the indent sites. The topographic images were used to derive a binary image mask with appropriately spaced patches that lay over each separate indentation site. This image was aligned with large, easily visible marker indents at the array boundaries [25]. The binary mask was overlaid on the corresponding compositional image and the mean gray level at each indentation site was determined from approximately 220 image pixels. A linear calibration curve was established to convert qBSE gray level values to equivalent MD using materials with known composition [24,29,30]. One SD was given in this paper for comparative purposes as the estimated standard uncertainty of the measurements.

### Polarized light microscopy

For PLM examination, small segments cut from all the specimens in this study were partially decalcified prior to embedding in paraffin wax, because complete decalcification could lead to collapse of the soft tissues that surrounded the mineral. Five μm thick sections were cut and stained with H&E and viewed on a Zeiss Axioplan 2 microscope (Carl Zeiss Microimaging, Thornwood, NY, USA) with a polarization to visualize collagen fibril and residual mineral organization, and photographed with an AxioCam HRc digital camera (Carl Zeiss Microimaging, Thornwood, NY, USA).

## Results

### FTIR-RM, SEM/SDD-EDS and μCT maps and images

**Figure 1:**
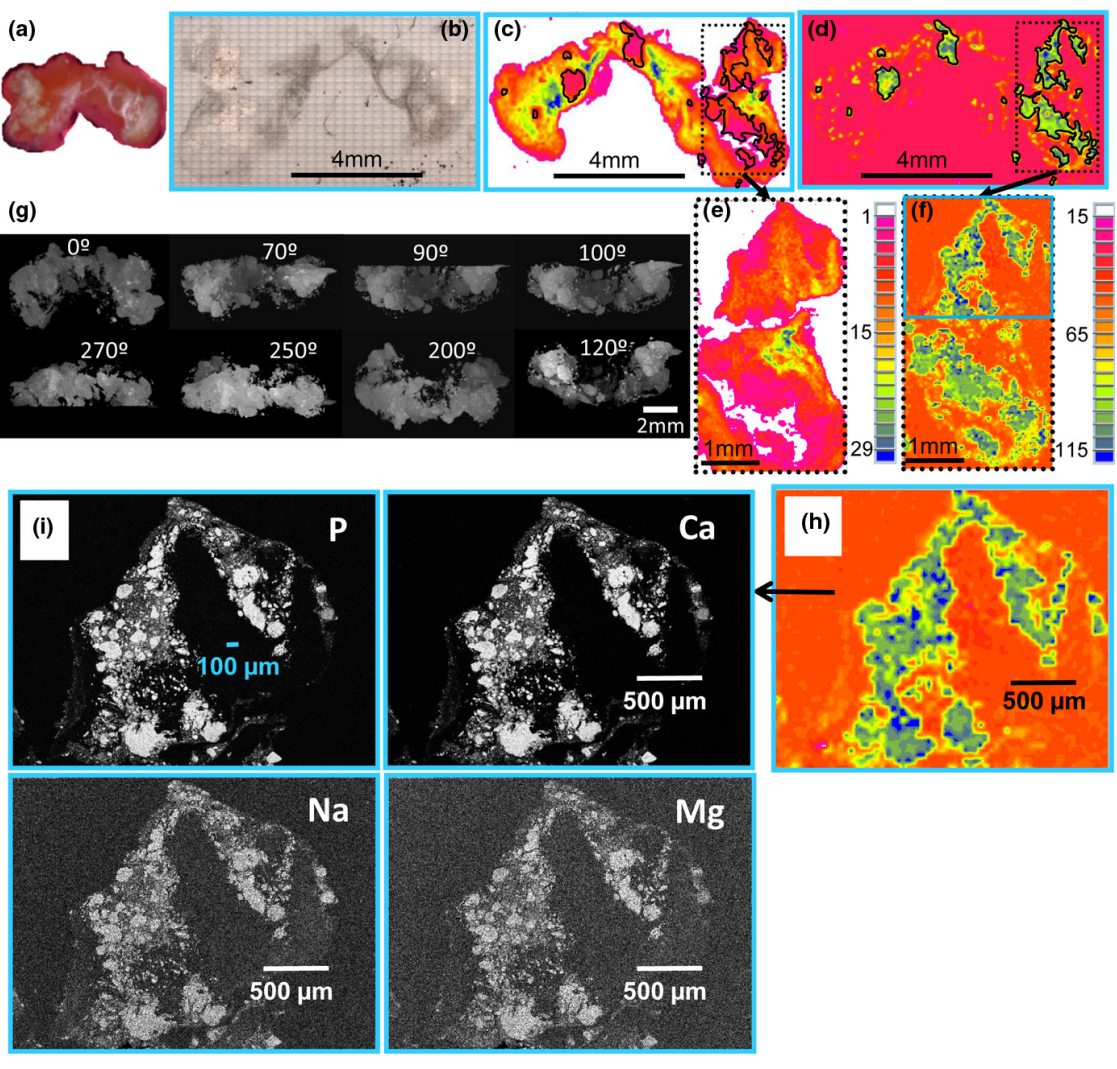
Specimen Calc2. **(a) **Optical image. **(b) **Visual map of the surface of the cut face after embedding and polishing. **(c and d) **Fourier Transform Infrared microspectroscopy in reflectance mode (FTIR-RM) protein and mineral maps (area under the amide and PO_4 _peaks, respectively) obtained from the whole specimen with 80 × 80 μm spatial resolution (SR). **(e and f) **FTIR-RM protein and mineral maps obtained from the boxed regions in (c) and (d) with 40 × 40 μm SR. **(g) **X-ray micro-computed tomography (μCT) 3D projection images rotated around the same plane that was mapped with FTIR-RM, silicon drift detector energy dispersive X-ray spectrometry (SEM/SDD-EDS) and quantitative backscattered electron imaging. **(h) **The cyan boxed region of the FTIR-RM mineral map (f) that was subsequently mapped with SEM/SDD-EDS. **(i) **SEM/SDD-EDS P, Ca, Na and Mg maps of the same region that was mapped before with FTIR-RM (h). A smaller size scale bar (100 μm) is shown in the P map to emphasize the small dimensions of many of the tiny calcified nodules.

**Figure 2:**
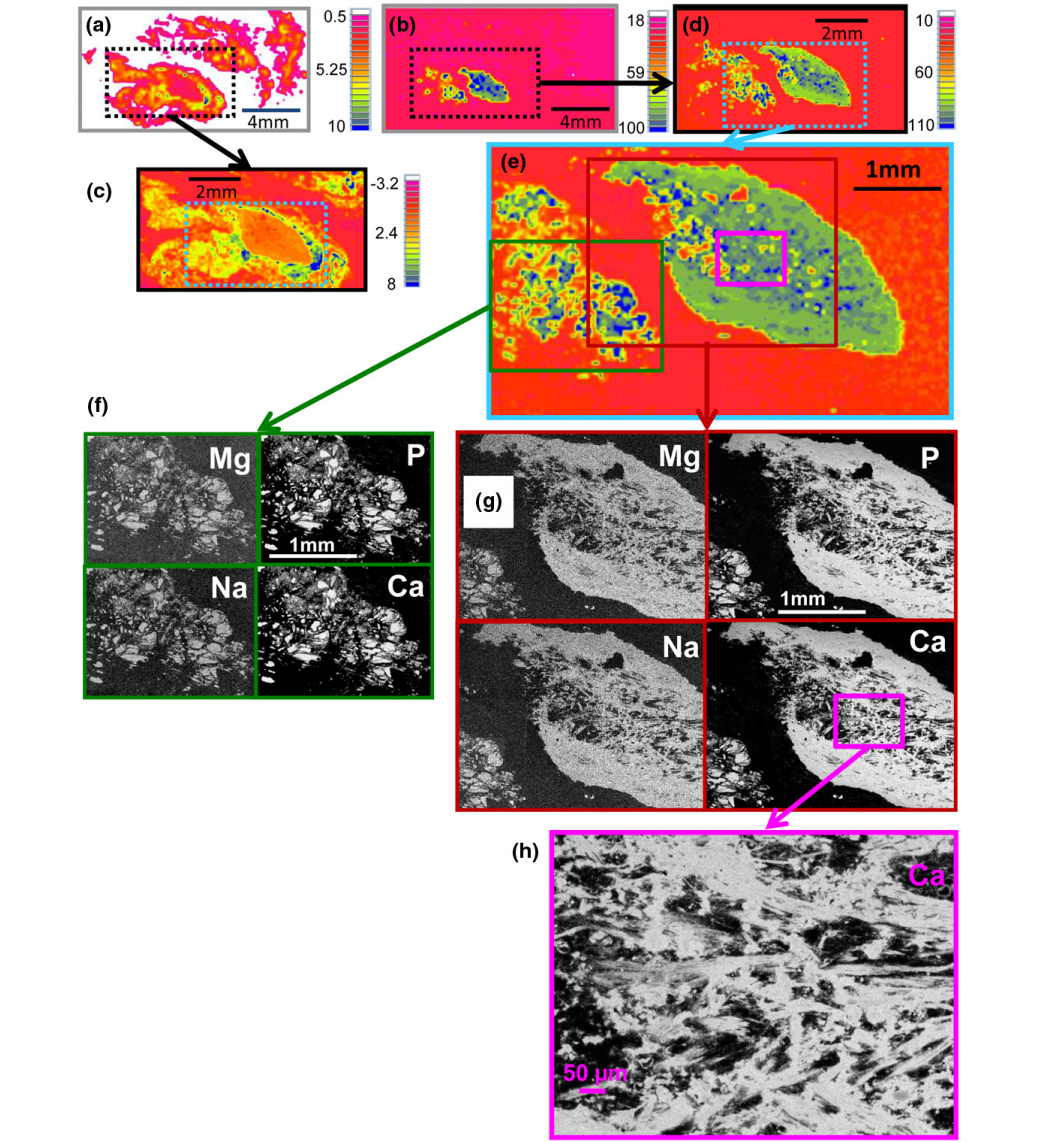
Specimen Calc1. **(a and b) **Fourier Transform Infrared microspectroscopy in reflectance mode (FTIR-RM) protein and mineral maps, respectively, obtained from the whole specimen with 200 × 200 μm spatial resolution (SR). **(c and d) **FTIR-RM protein and mineral maps obtained from the black boxed regions in (a) and (b) with 80 × 80 μm SR. **(e) **FTIR-RM mineral map obtained from the cyan boxed region in (d) with 40 × 40 μm SR. Blue is the largest area under the related peaks and red is the lowest in the FTIR-RM maps. The individual size scale bars appear on the images and the maps. **(f) **Silicon drift detector energy dispersive X-ray spectrometry (SEM/SDD-EDS) Mg, Na, P and Ca maps of the green boxed region in (e). **(g) **SEM/SDD-EDS Mg, Na, P and Ca maps of the brown boxed region in (e). The size scale bars in all the SEM/SDD-EDS maps in (f) and (g) are the same as in (e) (1 mm). **(h) **SEM/SDD-EDS Ca map of the purple boxed region in (e) and in the Ca map in (g), obtained with higher magnification.

**Figure 3:**
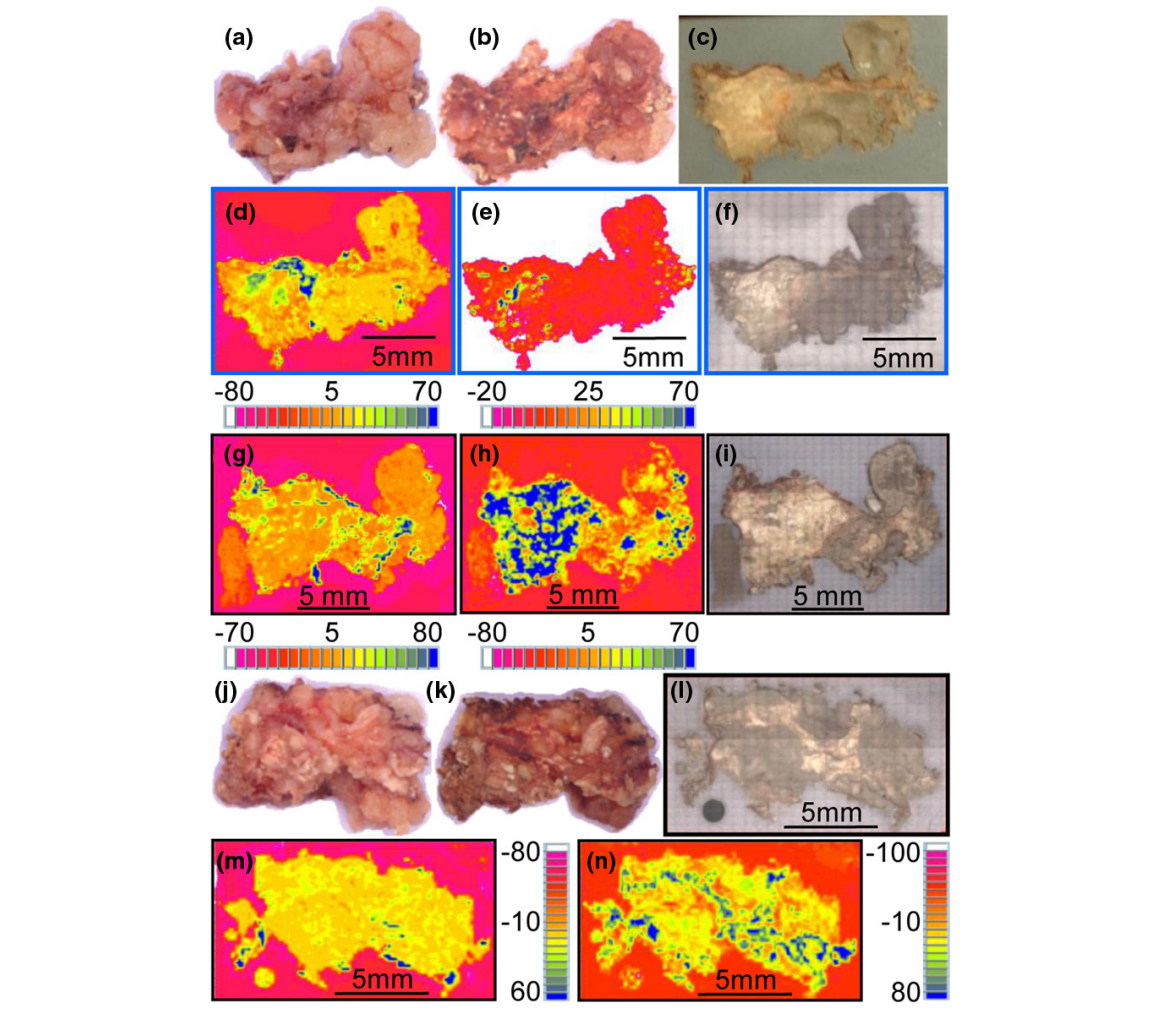
Specimen Calc10. **(a to c) **Images of the top of the specimen as received, after freeze drying, embedding and first polishing, respectively. **(d to f) **Fourier Transform Infrared microspectroscopy in reflectance mode (FTIR-RM) protein and mineral maps and visual map, respectively, of the first exposed plane. **(g to i) **FTIR-RM protein and mineral maps and visual map, respectively, of the second plane. **(j and k) **Images of the bottom of the specimen as received and after freeze drying, respectively. **(l to n) **Visual map and FTIR-RM protein and mineral maps, respectively, of the bottom after embedding and polishing. The distance between the second polished plane (i) and the shaved bottom (l) is 1.8 mm. All FTIR-RM maps were obtained with 180 × 180 μm spatial resolution (SR). Blue is the largest area under the related peaks and red is the lowest in the FTIR-RM maps. The individual size scale bars appear on the images and the maps.

The distribution of the calcified deposits within the tissue specimens differed among samples from different patients. In three specimens (Calc1, Calc2, and Calc10), calcified islands of various sizes were present in the cross sections surrounded by porous tissue (Figures 1, 2 and 3). The embedding material filled the pores while barely penetrating into the calcified regions (no carbonyl was detected in the FTIR-RM spectra obtained from highly calcified regions). More calcified nodules were seen in the mineral map obtained with the 40 × 40 μm spatial resolution (Figure 1f) than in the 80 × 80 μm map (Figure 1d), suggesting that many islands were smaller than 80 × 80 μm and may be even smaller than 40 × 40 μm, as was seen subsequently in the SEM/SDD-EDS Ca and P maps (Figure 1i). Selected μCT 3D projection images obtained from Calc2 that were rotated around the x-axis parallel to the cross section that was mapped first with FTIR-RM, are presented in Figure 1g. The straight cut top of the calcified nodules seen in the 90° image is the plane that was mapped with FTIR-RM, SEM/SDD-EDS and qBSE. The selected images representing different planes of the specimen showed that the calcified deposits, seen as scattered cut 2D islands in the cross section in the FTIR-RM and the visual maps, were continuous small scattered 3D nodules in the whole bulk of the mass, as seen especially in the Z cross sections (Figure 1g: 90°, 100° and 270°). The calcified islands in Calc1 (Figure 2) seen on the left of the mineral map in Figure 2e were small (≈ 40 × 40 μm to ≈ 300 × 300 μm) and most were mixed with the embedding material. They are seen in more details and with higher resolution in the SEM/SDD-EDS P and Ca maps (Figure 2f). The large calcified region on the right (≈ 3.5 × 2.2 mm) seems partially continuous with gaps and regions with higher degree of mineralization (blue regions, FTIR-RM mineral (PO_4_) map, Figure 2e), and surrounded by tissue (FTIR-RM proteins map, Figure 2c). The high resolution SEM/SDD-EDS P and Ca maps of this region (Figure 2g) present its edges as composed of condensed thin particles and its inside as thin separated elongated particles that appear to be thin needles under higher magnification (Figure 2h). Note that the areas with the highest calcification in the FTIR-RM phosphate (PO_4_) maps of Calc1 and Calc2 (Figures 1d and 2d) were seen as orange 'voids' with very little protein in the related amide maps (Figures 1c and 2c). There was no consistent positive or negative correlation between the mineral and the proteins in the PO_4 _and amide maps of Calc10 (Figure 3). Proteins were barely present on the left side of the amide map obtained after the second polishing (Figure 3g), while highly calcified spots (blue) were seen in the PO_4 _map (Figure 3h). Note also the presence of small scattered islands of calcified deposits in the cross sections of all three mapped planes across the Z direction of the specimen (Figures 3e, h and 3n). This indicates that most probably this nodular pattern existed in the whole specimen.

**Figure 4:**
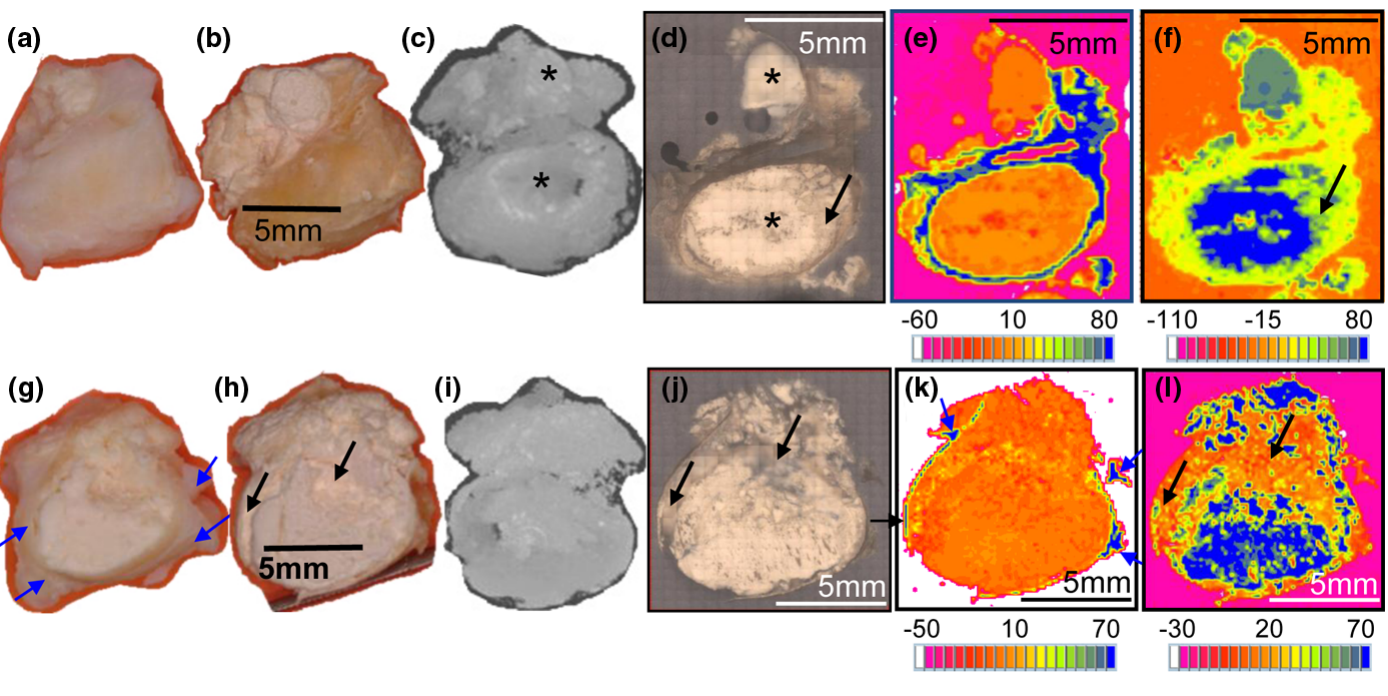
Specimen Calc7. **(a and b) **Images of the top of the specimen as received and after freeze drying, respectively. **(c to f) **X-ray micro-computed tomography (μCT) 3D projection image, visual and Fourier Transform Infrared microspectroscopy in reflectance mode (FTIR-RM) protein and mineral maps of the top, respectively, after embedding and polishing. * indicates the same exposed surfaces in the 3D μCT image (c) and the visual map (d). **(g and h) **Images of the cut cross section of the specimen as received and after freeze drying, respectively. **(i to l) **μCT 3D projection image, visual and FTIR-RM protein and mineral maps of the cut cross section, respectively, after embedding and polishing. The distance between the (d) polished top and the (j) polished cut cross section is 3.4 mm. All FTIR-RM maps were obtained with 180 × 180 μm spatial resolution (SR). Blue is the largest area under the related peaks and red is the lowest in the FTIR-RM maps. Blue arrows in G and K point to the surrounding tissue. The individual size scale bars appear on the images and the maps.

**Figure 5:**
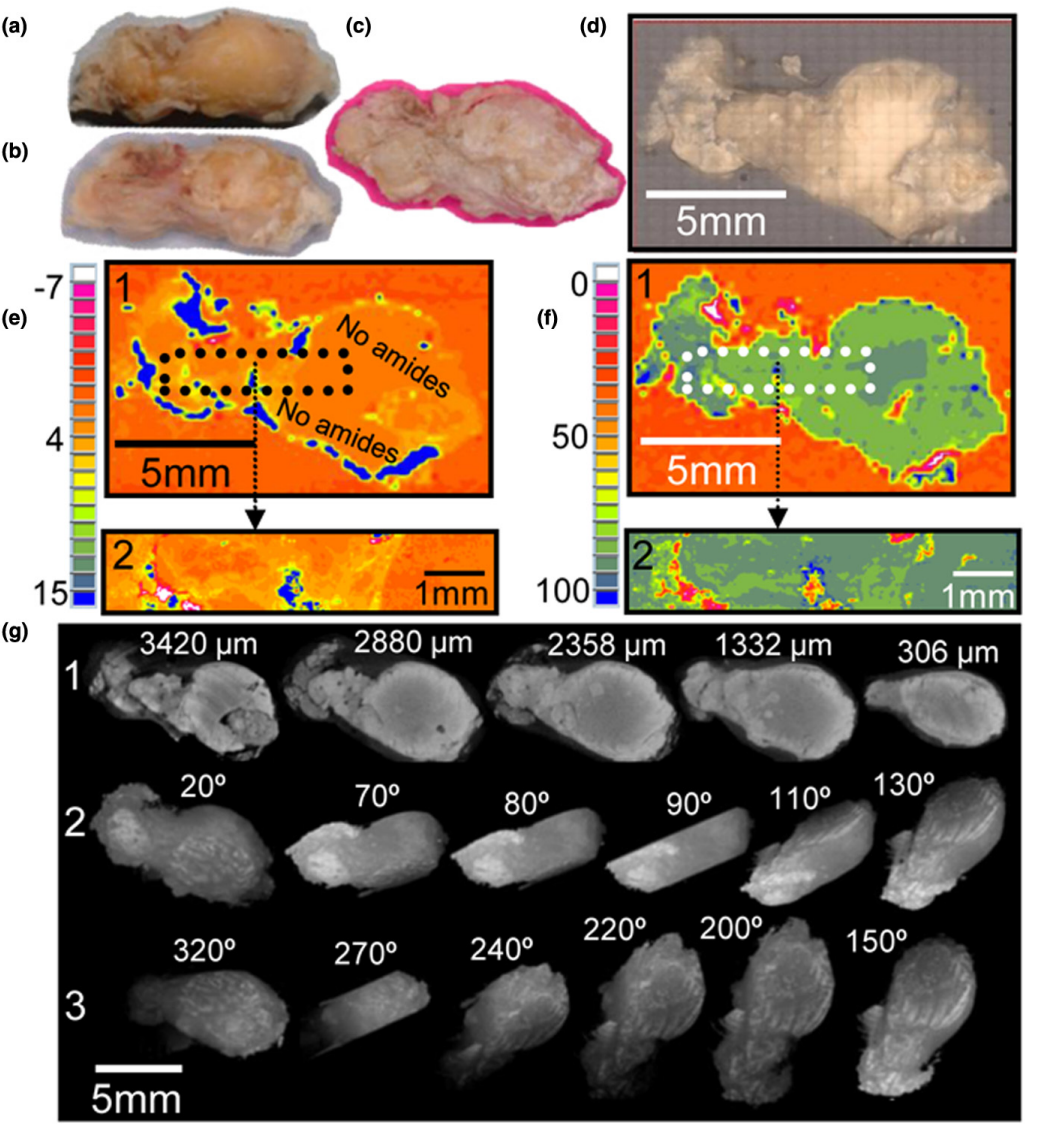
Specimen Calc8. **(a) **Bottom as received. **(b and c) **Cut face before and after freeze drying. **(d) **Visual map of the cut face after embedding and polishing. **(e) **Fourier Transform Infrared microspectroscopy in reflectance mode (FTIR-RM) protein maps obtained with **(panel 1) **200 × 200 μm and **(panel 2) **40 × 40 μm spatial resolution (SR). **(f) **FTIR-RM mineral maps obtained with **(panel 1) **200 × 200 μm and **(panel 2) **40 × 40 μm SR. Blue is the largest area under the related peaks and red is the lowest in the FTIR-RM maps. **(g, panel 1) **X-ray micro-computed tomography (μCT) 2D images obtained at various distances from the bottom of the embedded specimen. **(panels 2 and 3) **μCT 3D projection images rotated around the same plane as that mapped with the FTIR-RM. The individual size scale bars appear on the images and the maps.

In contrast, in Calc7 and Calc8 (Figures 4 and 5), a continuous mineral phase was present throughout the specimens, surrounded with tissue only at the edges. The bulk of Calc7 is continuously white in both the shaved top and the middle cut cross section (Figures 4d, h and 4j), but the surface could not be perfectly polished to the same plane because of its brittleness. Therefore, spectra that were obtained from rougher regions were distorted, and white calcified regions that are composed of continuous mineral were seen in the mineral maps with lighter colors than the blue of the highest intensity of the PO_4 _absorption (black arrows in Figures 4d, f, h, j and 4l). μCT 3D projection images (Figures 4c and 4i) confirmed the continuous calcification. Calc8 (Figure 5) was almost a continuous uniform, mineral rock without islands. No amides were present in the spectra of the dark orange regions inside the amide map (Figure 5e panel 1). These areas contained carbonate apatite without proteins. Small peaks of amides were present in spectra obtained from the lighter orange and yellow regions. The PO_4 _and amide maps of Calc8 that were acquired with 10 times higher spatial resolution (Figures 5e panel 2 and f panel 2) showed more details of the small protein regions (Figure 5e panel 2) and provided evidence that the carbonate apatite regions were fully continuous (Figure 5f panel 2). Note also that the blue spots in the amide map were red in the PO_4 _map. The calcification was virtually filling the entire specimen (blue-gray-green in the PO_4 _maps, Figure 5f panel 1 and 2), except for a few yellow-red spots that were gaps or tissue (blue in the amide maps, Figure 5e panels 1 and 2) surrounding the masses. Note also that most of the specimen is surrounded by tissue (blue, Figure 5e panel 1). Selected μCT 3D projection images of Calc8 rotated around the X-axis (parallel to the plane of the cross section that was mapped first with FTIR-RM) are shown in Figure 5g. The images represent different planes of the specimen and show that the calcification is continuous in all three dimensions.

### SEM/SDD-EDS analyses

The SEM/SDD-EDS maps of Ca, P, Mg and Na with lateral sampling resolution of 1 μm, were obtained from the highly calcified region of Calc2 (Figure 1i) and from two calcified regions in Calc1 (Figure 2g). These elemental maps show that the Na and Mg are in the same spots as the P and Ca, indicating that they precipitated together with the Ca and P forming one mineral phase composed of carbonate apatite (FTIR-RM) containing Mg and Na and trace amounts of Cl, S and Al. The relative concentrations (weight %, wt%) of Ca, P, Na, Mg, Cl, Al and S and the Ca/P molar ratios are presented in Table 2. Note that the Ca/P ratios are about the same between the two regions of Calc1 and between Calc1 and Calc2. Note also that the EDS maps show the calcified deposits as small islands (about 20 to 300 μm) in Calc2 and even smaller islands in Calc1.

**Table 2:** qBSE, FTIR-RM, SEM/SDD-EDS and XRD results obtained from various calcified tissues, calcinosis samples and synthetic apatites

*Sample/Analysis*									
**qBSE**		**MD, g/ml**							
Enamel		**1**: 2.98 ± 0.10;				**2**: 2.99 ± 0.09			
Dentin		**1**: 2.46 ± 0.44;				**2**: 2.46 ± 0.32			
Calcified cartilage		**1**: 2.36 ± 0.67;				**2**: 2.50 ± 0.81			
Bone		**1**: 2.14 ± 0.55;				**2**: 2.19 ± 0.55			
Calc1		**W**: 2.65 ± 0.74							
Calc2		**W**: 2.51 ± 0.74							
Calc2		**CE 1**: 2.68 ± 0.55;				**2**: 2.53 ± 0.75			
Calc2		**3**: 2.23 ± 0.79;				4*: 2.58 ± 0.54;			
		**5**: 2.66 ± 0.60							
Calc3**		**W**: 2.52 ± 0.92 (377);				**HC**: 2.69 ± 0.11 (245)			
Calc4**		**W**: 2.400 ± 1.09 (325);				**HC**: 2.66 ± 0.21 (147)			
									
**FTIR-RM**		**PO_4_/Am I**		**AmI/PO_4_**		**Calc/Bone**			
				**%**		**%**			
Dentin, Fig 7a		10.5		9.6					
Bone, Fig 7a		7.4		13.5					
Calc1, High Prot		11.8		8.5		62.7			
Calc1, Fig 7a		19.4		5.2		38.1			
Calc2, Fig 7a		27.8		3.6		26.6			
Calc10, Fig 7a		51.0		2.0		14.5			
Calc7, Fig 7a		30.6		3.3		24.2			
Calc8, Fig 7a		162.3		0.6		4.6			
									
**SEM/SDD-EDS**		**Ca**	**P**	**Ca/P**	**Mg**	**Na**	**Cl**	**S**	**Al**
		%	%	MR	%	%	%	%	%
Calc1-1:	Average	39.4	18.3	1.66	0.60	1.28	0.07	0.01	0.07
	SD	0.02	0.02		0.01	0.01	0.01	0.005	0.01
Calc1-2:	Average	39.8	18.2	1.69	0.62	1.07	0.05	0.02	0.07
	SD	0.02	0.02		0.01	0.01	0.01	0.005	0.01
Calc2:	Average	39.1	18.5	1.63	0.53	1.16	0.11	0.06	0.06
	SD	0.02	0.02		0.01	0.01	0.01	0.01	0.01
									
**XRD**		**1/FWHM**				**Crystallinity (Relative to Bone)**			
Phys Apatite		2.50				1.30			
Dentin		1.99				1.03			
Bone		1.93							
Calc7-Powder		3.76				1.95			
Calc7-Polished		4.10				2.13			
Calc8-Polished		4.85				2.52			
Enamel		3.53				1.83			
OHAp		5.95				3.09			

**W **= **w**hole image; **CE **= **c**alcified element; * Figure 5f; ** qBSE images in Figure 7c; **HC **= highly calcified regions (> 2.60 g/ml).

n = 1024 pixels for qBSE analyses of all calcified tissues and Calc1 and Calc2 images.

% = weight %; MR = molar ratio.

FTIR-RM = Fourier Transform Infrared microspectroscopy in reflectance mode; FWHM = full width at one half the maximum height; qBSE = quantitative backscattered electron; SEM/SDD-EDS = scanning electron microscopy/silicon drift detector energy dispersive X-ray spectrometry; XRD = X-ray diffraction.

### qBSE imaging

**Figure 6:**
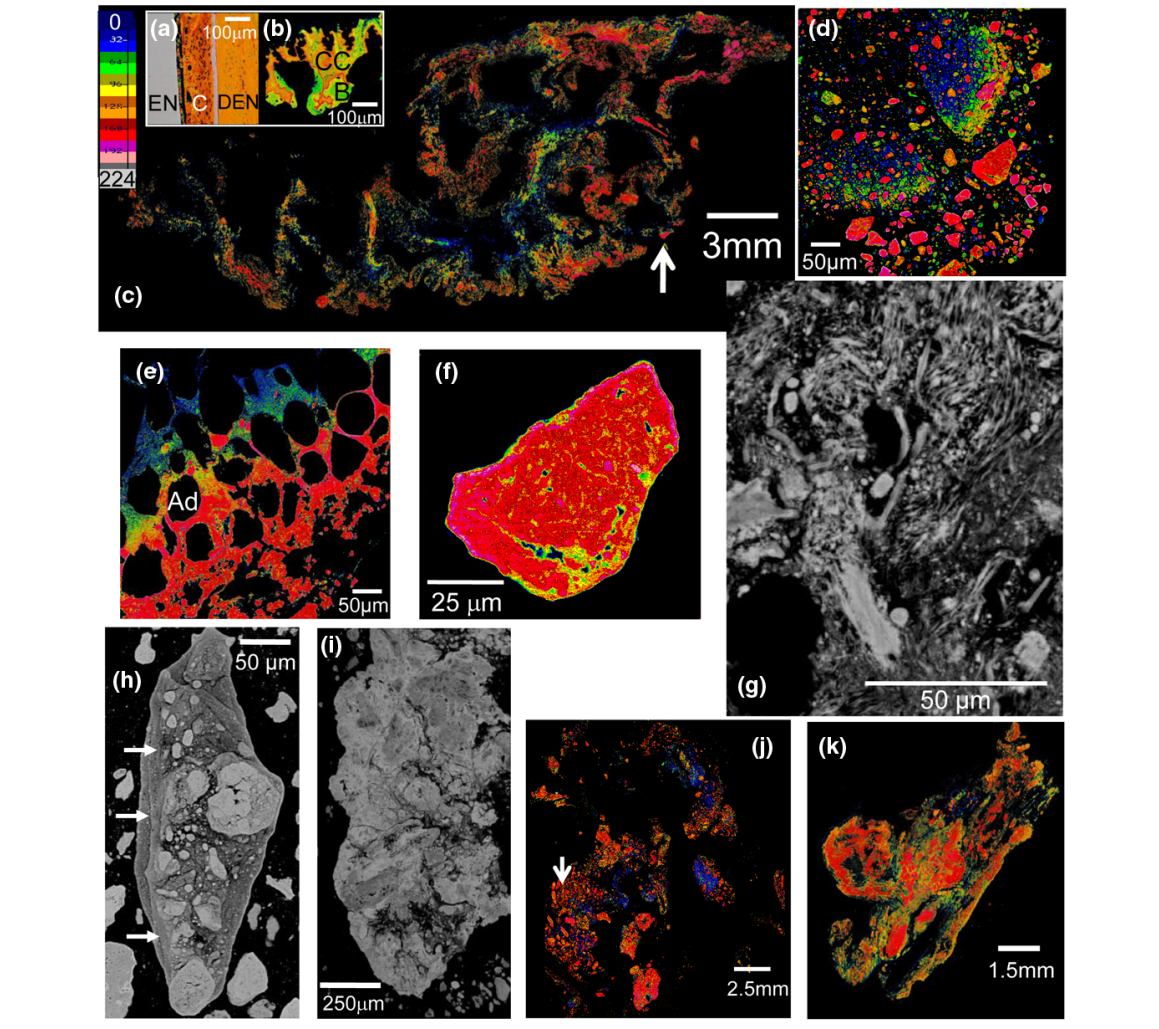
qBSE images. **(a) **Normal rabbit molar tooth. En = enamel; C = cementum; Den = dentin. **(b) **Human iliac crest. CC = calcified cartilage; B = bone. **(c) **Tiled image of the whole cross section of Calc3. The arrow indicates the region from where the nanoindentation measurements were obtained. **(d and e) **High magnification of vastly mineralized areas in specimens Calc2 and Calc3. The 'black holes' where no mineral was deposited are adipocyte 'fat'(Ad). **(f) **Higher magnification of the largest particle in (d). **(g) **High magnification of poorly mineralized region in Calc3. The arrow indicates collagen fiber bundles and the asterisk indicates smooth muscles. **(h and i) **Highly mineralized nodules in Calc 4. The arrows in h are pointed towards incremental growth lines that indicate waves of calcification. **(j and k) **Tiled images of the whole cross sections of Calc4 and Calc6. The mineral density (MD) is the lowest in the dark blue regions and the highest in the light gray regions, as in the enamel in (a) in all color images. A lighter gray shade represents higher MD in the gray scale images. The individual size scale bars appear on each image.

To visualize the distribution of mineralization densities, a pseudo-color look up table (Figure 6, top left panel) with 16 equal ranges was utilized. Normal rabbit molar tooth with coronal cementum, enamel and dentin (Figure 6a) and human iliac crest (9-year-old female) displaying islands of calcified cartilage and bone (Figure 6b) were examined simultaneously for comparison to the calcinosis specimens. As was typically seen in all specimens examined by qBSE (Calc1, Calc2, Calc3, Calc4 and Calc6) and illustrated in Calc3, Calc4 and Calc6 at low magnification (Figures 6c, j and 6k), the calcified areas were not continuous and displayed a great deal of heterogeneity in the degree of mineralization, ranging from poorly mineralized (blue) to highly mineralized (red) areas. At higher magnification of poorly mineralized areas, there was a predominance of fibers with varying morphological appearance (as can be seen in Calc3, Figure 6g). Regions such as these in the specimens with island morphology that were mapped with FTIR-RM (Calc1, Calc2 and Calc8) were composed of amides that are the main functional groups of all proteins and soft tissues. In more mineralized areas, small, more highly mineralized regions (red particles) were seen embedded in a background of less mineralized material (blue-green-yellow regions, Figure 6d). A gradient in the MD of this background material from lower (blue) to higher (red) is seen also in Figure 6e (Calc3). The black holes in Figure 6e represent the prior location of adipocyte fat (itself removed by defatting during ethanolic dehydration and methacrylate monomer substitution). Zooming in on large highly mineralized nodules revealed substructures (Figures 6f, h and 6i) and incremental growth lines (waves of calcification, arrows in Figure 6h). Reviewing the montage images of Calc3, Calc4 and Calc6 (Figures 6c, j and 6k) reveals that these specimens are composed of scattered mineralized islands. The arrow in Figure 6j points to the large agglomerate shown in Figure 6h.

To make a direct comparison of the calcinosis specimens with normal calcified tissues, the qBSE image histograms from selected regions were compared with two regions from each of rabbit molar dentin and enamel and human iliac crest bone and calcified cartilage. The MD values are presented in Table 2. Typically, the mineralized regions had higher MD than bone, and often higher than dentin and calcified cartilage, but less than enamel.

### Polarized light microscopy

**Figure 7:**
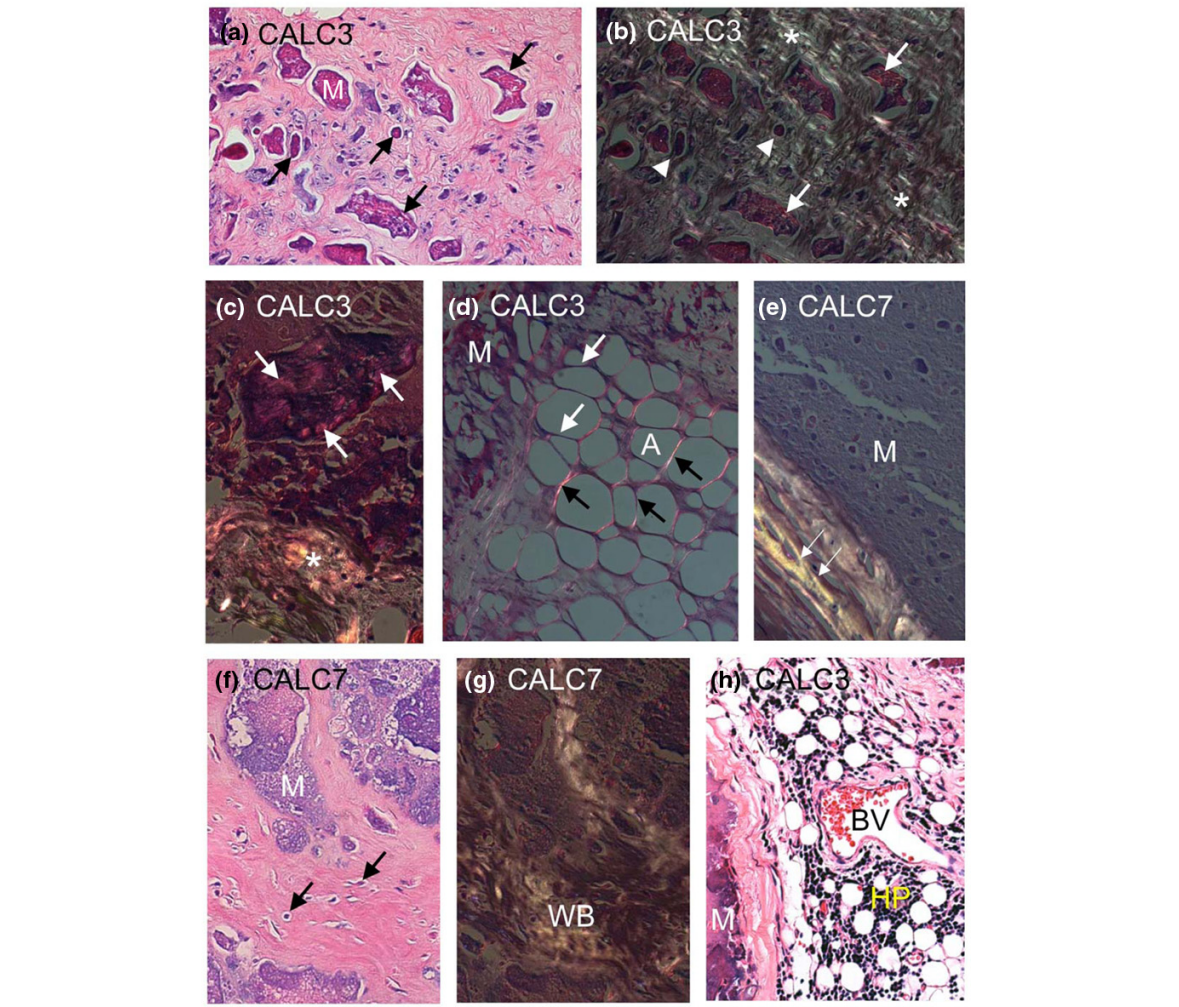
Bright field and polarized light microscopy. **(a, f and h) **Bright field and **(b to e and g) **polarized light microscopy of **(a to d and h) **Calc3 and **(e to g) **Calc7. a and b are the same fields showing small mineralized nodules (arrows) interspersed within a collagenous matrix as illuminated by polarized light (asterisks in b and c). In some cases, the nodules are devoid of collagen (arrow heads in b), and in other cases, wisps of collagen can be detected by the polarized light (arrows in b and c). In other areas (d), adipocytes (A), which are usually surrounded by a delicate lattice visualized by polarized light (black arrows), a lack of polarization surrounding adipocytes (white arrows) near areas of mineral (M) is noted. In areas of mineral that are completely encapsulated by a collagenous matrix (e, white arrows), mineral was found to be completely devoid of collagen as demonstrated by the lack of polarization. f and g are the same fields. Areas of woven bone-like material (WB in g) were found within acellular lakes of mineral (M), with cells embedded within the matrix (arrows in f). Occasionally, hematopoietic tissue (HP in h) appeared in close proximity to blood vessels (BV) and mineral (M).

Examination of almost completely decalcified paraffin-embedded sections stained with H&E with bright field light microscopy and PLM revealed several interesting features. In all but one sample (Calc10), large lakes of acellular material were noted in the bright field images (arrows in Figure 7a, M in Figures 7a, f and 6h, stained light purple), which contained numerous smaller nodular structures of darker staining material. These acellular lakes were devoid of collagen for the most part, but surrounded by a collagen-rich connective tissue as demonstrated by PLM (Figure 7e, white arrows). In some cases, the nodules were devoid of collagen (arrow heads in Figure 7b), and in other cases, wisps of collagen could be detected by the polarized light (arrows in Figures 7b and 7c). In samples from Calc1, Calc2, Calc3 and Calc10 that also contained smaller mineralized nodules (Figure 7b, arrows), collagen fibers were seen interspersed with mineralizing material by polarized light (Figures 7b and 7c, asterisks). In other areas (Figure 7d), adipocytes (A), which are usually surrounded by a delicate lattice of collagen visualized by polarized light (black arrows), the matrix surrounding them in mineralized areas, was devoid of collagen as noted by a lack of polarization surrounding adipocytes (white arrows, Figure 7d) near areas of mineral (M), which is uncharacteristic of normal adipose tissue. In areas of mineral that are completely encapsulated by a collagenous matrix (Figure 7e, white arrows), mineral was found to be completely devoid of collagen as demonstrated by the lack of polarization. Very rarely areas with a histological appearance of woven bone (WB in Figure 7g) were found within acellular lakes of mineral (M) with embedded cells as demonstrated by bright field microscopy (Figure 7f, arrows) and by polarized light (Figure 7g). In addition, in two samples (Calc3 and Calc6), small areas of hematopoietic marrow (HP, Figure 7h) surrounding by blood vessels (BV, Figure 7h) were seen in close proximity to the large mineral deposits (M, Figure 7h).

In summary, there was no evidence of collagen in the vast majority of the acellular lakes of mineral as indicated by the lack of illumination by polarized light images (Figure 7). Only in small nodules on the periphery (presumably where the crystal is growing) small wisps of collagen could be seen in the nodules.

### FTIR-RM spectra

**Figure 8:**
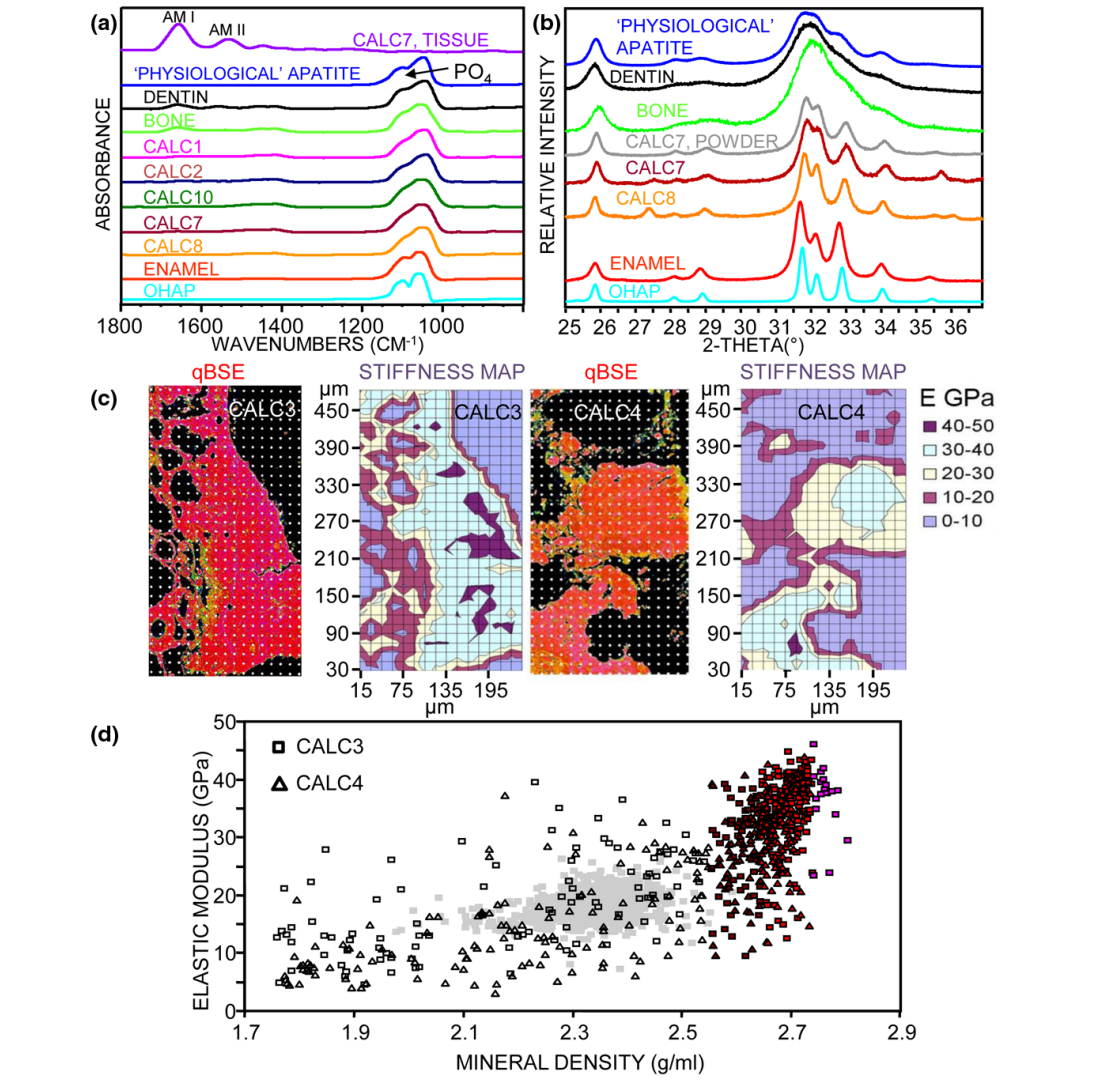
FTIR-RM spectra, XRD patterns, qBSE and stiffness maps and elastic modulus results. **(a) **Representative Fourier Transform Infrared microspectroscopy in reflectance mode (FTIR-RM) spectra extracted from the maps of Calc1, Calc2, Calc7, Calc8 and Calc10 compared with these of bone, dentin and enamel specimens, and to pressed pellets of physiological apatite and highly crystallized hydroxyapatite. **(b) **X-ray diffraction (XRD) patterns of powder from Calc7, of the cross sections of Calc7 and Calc8 compared with those obtained from powders of bone, dentin, enamel, physiological apatite and highly crystallized hydroxyapatite. **(c) **quantitative backscattered electron imaging (qBSE) images and matching stiffness maps obtained from Calc3 and Calc4. The darkest purple represents the highest stiffness and the lightest purple the lowest. **(d) **Degree of stiffness (elastic modulus) versus the mineral density (MD) measured at the same locations for Calc3 (square) and Calc4 (triangle). Colored data points correspond to data from the dark red, red and pink areas of the qBSE images in c. Hollow symbols correspond to all of the other colored areas in c.

All the spectra obtained from distinct calcified regions without surrounding tissue and/or embedding material, from the five specimens that were mapped with FTIR-RM (Table 1), contained PO_4 _peaks typical of apatite and similar to that of bone; carbonate peaks similar to those present in bone; and small amide peaks (of proteins and/or collagen) with much lower intensity than in bone (Table 2). The representative spectra shown in Figure 8a contained 5% to 38% of that present in bone. Only a few spectra from Calc1 had higher protein amounts (71% relative to bone in the spectrum reported in Table 2). Some spectra in dark gray-blue regions in the PO_4 _map of Calc8 (Figure 5f) had only traces of amides. The FTIR-RM spectra indicated that the mineral that formed in all five calcinosis specimens analyzed with FTIR-RM was carbonate apatite, with small amounts of proteins, although retrieved from patients with various calcinosis conditions, ages, disease duration and treatment (Table 1). Note that similar spectra were obtained from all specimens, despite using two different embedding materials and polishing regimens.

### X-ray diffraction

The only mineral detected by XRD in the powder obtained from Calc7 was apatite (Figure 8b). The XRD pattern obtained from the whole polished cross section of Calc7 was similar to that of the powder and confirmed that there are no other calcium phosphate phases or inorganic compounds than apatite present in the whole specimen (Figure 8b). These patterns, as well as that obtained from the whole cross section of Calc8, show that the apatite in the calcinosis specimens is much more crystallized than that present in bone and dentin, and closer to enamel (Figure 8b). Note that sharper and more resolved peaks represent higher crystallinity [20]. The related 1/FWHM and their relative values to these of bone (Table 2) indicate that the crystallinity of the apatite present in Calc7 and Calc8 is about 2 and 2.5 times higher than that of bone and dentin, slightly higher than enamel and lower than the highly crystallized solid-state thermally prepared hydroxyapatite [16].

### Nanoindentation measurements

The qBSE images obtained from Calc3 and Calc4 with the overlaid mask marking the indents location along with the matching stiffness maps based on 15 μm measurement array are shown in Figure 8c. The arrow in Figure 6c indicates the region in Calc3 from where the nanoindentation measurements were obtained. Site matched values of tissue stiffness (elastic modulus) and MD from Calc3 and Calc4 are cross-plotted in Figure 8d. Indentation sites that were located in areas that did not contain calcified deposits and/or contained low mineral (black in the qBSE images of Figure 8c) were not included in Figure 8d. Data from normal human bone [25] are overlain in gray for comparison. The majority of the data in Figure 8d lie at mineral density values greater than 2.55 g/ml (colored dark red, red or pink) and display a linear correlation (R^2 ^= 0.58) between stiffness and the higher degrees of mineralization as measured by qBSE in larger dense patches (Figure 8d). The stiffness results of the indents of Calc3 and Calc4 that were obtained from mineralized regions in the qBSE images were 29.9 ± 9.5 GPa (357 indents) and 25.6 ± 8.2 GPa (253 indents) respectively. Stiffness values below 10 GPa obtained from indents that were performed on black empty regions in the qBSE images and seen as light purple regions (0 to 10 GPa) in the stiffness maps in Figure 8c, were excluded from the average. The average of the high 'concentration' of stiffness values of Calc3 and Calc4 at MD greater than 2.55 g/ml (colored data points in Figure 8d) was 33.7 ± 6.9 GPa (263 indents) and 29.0 ± 6.7 GPa (168 indents), respectively.

## Discussion

Our analyses revealed that the mineral present in the calcinosis lesions of juvenile and adult myositis patients consists of carbonate apatite and not hydroxyapatite or fluoroapatite, as it was referred to in earlier case reports of surgically removed calcinosis specimens from patients with calcinosis cutis, JDM and DM, using XRD [9-12]. Nielsen and colleagues [10] stated that the accuracy of the selected area diffraction method used in their study was not sufficient to distinguish between hydroxyapatite and or fluoroapatite. The mineralized regions also contained small amounts of proteins that were significantly lower than those present in bone (Table 2), as determined in the FTIR-RM analyses. A higher mineral-to-protein ratio than that of bone has been previously reported in deposits from five JDM patients, using FTIR spectroscopy on powders from ground specimens and FTIR imaging [8] that was performed on small 2 to 4 μm sections, while we mapped the whole specimens. The Ca/P molar ratios that were determined in two regions of Calc1 and one region of Calc2 with SEM/SDD-EDS (Table 2) were close to that of stoichiometric hydroxyapatite (1.67). Those results add important new information about the composition of the calcified nodules in this clinical disorder.

XRD performed on two of the five specimens that were analyzed with FTIR-RM (Calc7 and Calc8) showed that the mineral is highly crystallized apatite. The crystallographic results (XRD) corroborate those of the elemental analysis (SEM/SDD-EDS). Similar FTIR spectra were obtained from calcified nodules present in the five different specimens that were subjected to FTIR-RM and Ca/P ratios obtained with SEM/SDD-EDS from two of these five specimens were very similar between the two specimens and between the two large calcified regions in the same specimen (Calc1). These findings indicate that although the deposits differ in their sizes, internal distribution and microstructure, their chemical composition is the same.

The FTIR-RM and XRD data in this study, in confirming the carbonated apatitic nature of the mineral phase - which is the nature of the mineral in normal calcifying tissues (enamel, dentin, cementum, cartilage and bone) - indicate that it is safe to make direct assumptions about the mineral content from comparison of the qBSE signal levels from these different tissues. The findings that the crystallinity (by XRD) of the apatite was more than two times higher than that of bone and dentin and closer to that of enamel, that the Ca/P ratios (by SEM/SDD-EDS) are close to that of stoichiometric hydroxyapatite, and that the deposits contained significantly less organic matrix than bone and dentin (5% to 38% relative to that present in bone in representative spots in specimens obtained from various patients, by FTIR-RM), correlate with the results obtained from the qBSE imaging that the mineral density of the calcified areas was much higher than bone, often higher than dentin and the more highly mineralized regions within calcified cartilage, and closer to that of enamel (Table 2). This also correlates with the nanoindentation measurements, in that the stiffness values of the highly mineralized regions in the analyzed calcinosis samples (33.7 ± 6.9 and 29.0 ± 6.7 GPa) are greater than human dentin (20 GPa to 27 GPa) [33] and normal human bone (16.8 ± 2.8 GPa) [34] and below those of enamel (70 GPa to 100 GPa) [35]. This also correlates with the histological findings of an absence of collagen (the main component of the organic matrix in bone and dentin) in highly mineralized areas.

Earlier microscopic examinations support our PLM findings of the scarce presence of collagen inside the calcified regions. Paegle [9] proposed that the random orientation of the long axes of the crystals, their large size and lack of association with collagen and elastin, suggested that the crystallization occurred in the extracellular spaces, mitigating against the direct role of collagen in mineral deposition in calcinosis coutis. Nielsen and colleagues [10] claimed that they demonstrated with electron microscopy (EM) that only elastic fibers calcified and not collagen in the case of a five-year-old girl suffering from JDM and universal calcinosis. Kawakami and colleagues [11] reported that the calcified deposits formed in the site of fibrinoid degeneration and were located partly on collagen fibers, but mainly on cell debris in foci of degeneration. Globular and/or membranous structures (originated from the degenerated cells of the stroma) that were observed in these calcified regions, were hypothesized to be concerned with initial calcification in this case of calcinosis universalis associated with dermatomyositis. Landis [36] showed that in an adult DM case, the long axes of the mineral rods followed the long axes of associated collagen, but without a definitive structural relation with the collagen period.

Our study demonstrates the advantages of FTIR-RM as a non-destructive method capable of mapping large thick specimens (as big as 120 × 25 × 14 mm). Full DM specimens (as large as 14 × 10 mm) were fully mapped at various planes and subsequently could be remounted and analyzed by XRD, μCT and SEM/SDD-EDS, while the only published results of FTIR imaging of a JDM specimen presented an FTIR image obtained from an area of 312 × 375 μm of a 2 to 4 μm thin section [8]. In our study, the XRD analyses performed at the same areas on the same specimens corroborated the FTIR-RM findings, confirming that the only inorganic compound present in the DM deposits was indeed apatite and that it was much more crystallized than bone. The FTIR-RM measurements in our study were performed in a manner to resolve the spatial distribution of both the mineral and the organic matrix, as well as the distribution of the deposits within the specimens. In this way, we fully determined the composition, distribution and spatial orientation of the mineral in the cross sections of the whole specimens, as well as in various depths of the bulk. The μCT performed on three specimens confirmed that the distribution of the calcified deposits seen in the 2D FTIR-RM maps represented very well the 3D network of the deposits.

This is the first report of the use of SEM/SDD-EDS to perform detailed elemental analyses and mapping on DM deposits. The similarity of all the nodules (including the tiny particles) in the Ca, P, Na and Mg maps (Figures 1i, 2f and 2g) indicate that these elements precipitated together in the same spots, forming one homogenous mineral composed of carbonate apatite (FTIR-RM, XRD) containing Na, Mg and trace amounts of Cl, Al and S. Earlier X-ray micro analysis study detected Ca, P and small amounts of Cl and S in the deposits of a JDM and universal calcinosis case [10]. No values for the amounts of these elements were reported. In addition, an electron probe microanalysis demonstrated that the calcified deposits in a case of calcinosis universalis associated with DM [11] consisted mainly of Ca and P and that stromal matrices contained traces of Ca and P and small amount of S. The S peak existed mainly in the stromal tissues. No values for the amounts of these elements were reported either, while the two deposits obtained from JDM and JPM patients that we mapped with SEM/SDD-EDS, consisted mainly from Ca, P and O with Na and Mg as minor elements and trace amounts of Cl, Al and S, and their relative amounts were determined (Table 2). The finding of Mg in these DM calcinosis deposits is novel and of interest, as Mg is a known physiological mineralization inhibitor [37].

Our XRD analyses were performed on particles that were scraped from the white chalky part of a native unprocessed specimen (Calc7) and on the highly mineralized regions (white parts) of the cross sections of embedded and polished whole native specimens (Calc7 and Calc8) and not on fully ground specimens that may include proteins and other organic materials from the surrounding tissue, that are not necessarily part of the calcified regions as was performed in earlier studies [8,11]. Our study shows patterns of apatite that is much more crystallized than bone and dentin, with highly resolved peaks in the 30 to 34° 2θ region. A published XRD pattern [12] obtained from a 0.5 × 0.5 mm area of a JDM sample with synchrotron transmission XRD also showed more resolved peaks than in bone, while XRD patterns similar to bone with c-axis length in the same range as in bone were reported in fat and muscle samples from a JDM patient in another study [8]. The quantitative XRD results obtained in our study (Table 2) cannot be compared directly to these of Stock and colleagues [12], because the width of the 002 peak was not reported in that publication. Crystallite sizes of 220 Å and 240 Å were estimated for one JDM sample and for a trabecular bone sample (respectively) using the FWHM of the 002 peak in Scherrer's equation [12].

This is the first report of the use of qBSE imaging to study the detailed morphology and mineral density of soft tissue calcification with such high resolution. A great advantage of the BSE block face approach to tissue analysis concerns its depth of resolution. The information depth is of the order of 0.5 μm [38], and the lateral resolution can be better than 0.1 μm depending on the pixel size chosen. Thus, we have a resolution that is orders of magnitude better than X-ray microanalysis and X-ray imaging. We have about 0.5 μm electron optical section in an area limited only by the block dimensions. Further, the 'section' in a well embedded PMMA block face is intact, because it is the intact tissue in the residual block face, whereas any physical section will be incomplete and deformed, particularly if it contains tiny calcified elements. These exceptional specifications of the qBSE resulted in information about incremental growth lines and substructural details (Figure 6h). Another advantage is that the same surface of these blocks can be mapped with FTIR-RM without any additional treatment or alteration, as we did when Calc1 and Calc2 blocks were imaged first with qBSE, subsequently mapped with FTIR-RM and SEM/SDD-EDS, and then scanned with μCT that supplied the internal structure of the deposits inside the specimen (Calc2).

Comparing the FTIR-RM and SEM/SDD-EDS maps and the μCT and qBSE images with the clinical data (Table 1) reveals a correlation between the duration of calcinosis and the morphological distribution of the mineral in the specimens. The duration of the presence of calcinosis in samples Calc1 (FTIR-RM, qBSE, SEM/SDD-EDS), Calc2 (FTIR-RM, μCT, qBSE, SEM/SDD-EDS), Calc3 (qBSE), Calc6 (qBSE) and Calc10 (FTIR-RM), where islands of mineral are seen, is less than 12 months, and the patients' illness duration was six years or less. The illness duration for the patient of Calc4, that also exhibits the islands morphology, was 4.9 years while the duration of the calcinosis was at least three years. The patients with specimens of homogeneous and continuous mineral that filled the entire 'tissue sac' (Calc7 (FTIR-RM, μCT) and Calc8 (FTIR-RM, μCT)) had illness for 12 and 30 year (respectively) and calcinosis in these locations for at least 12 and 27 years. A relation between the duration of treatment and the mineral distribution was observed in a smaller study using μCT [12], in which continuous uniform mineralization was observed in a specimen removed from a patient who received treatment for only two months (9.5 years after the diagnosis of JDM), while smaller variable mineral blocks were observed in two other specimens removed from patients treated for 6.5 years and 7.2 years (6.7 years and 7.5 years after onset of JDM, respectively). Stock and colleagues [12] proposed possible origins for the inhomogeneous vs. uniform structure of JDM calcifications, including differences in duration of untreated inflammation, in the TNFα-308A polymorphism, and in physical constraints at the calcification site. That proposed relation between the morphology and location where the growing mass was confined or free to expand, is questionable from our larger study. Samples with the island morphology (Calc1 and Calc2) and other with continuous morphology (Calc7 and Calc8) were all from confined locations (elbow, toe, and shoulder).

The steep gradient pattern of mineral density observed in the background material in Figures 6d and 6e (seen as changing colours from blue to yellow-red in the top left of the images), and the solidly red regions in the right parts of the images, suggest that the mineralization process occurred in at least two stages, first with the formation of small mineralized nodules, followed by a wave of mineralization that incorporated such nodules into larger mineralized structures. The sizes of the nodules range from a few μm to about 100 μm (e.g., the largest particle in Figure 6d). Note that many islands of this size range were seen in the FTIR-RM mineral map of the same specimen (Calc2) when mapped with 40 × 40 μm spatial resolution (Figure 1f) and in the SEM/SDD-EDS P, Ca, Na and Mg maps (Figure 1i). Even highly mineralized regions displayed substructures (Figures 6h and 6i), and occasionally incremental growth lines, suggesting a resting period between rounds of mineralization, or variations in the rate of mineralization (arrows in Figure 6h). The gradient of background level mineralization seen in this study by FTIR-RM, μCT and qBSE is of considerable interest in understanding the biology of this disease. The morphology shows that widespread mineralization of large numbers of separate small zones or patches may occur, as if there were equal numbers of niduses at which the process commenced, perhaps within a short span of time. The small and highly mineralized patches may later become integrated via a general spread of mineralization (as seen also in the SEM/SDD-EDS elemental maps of the right region of Calc1; Figure 2g). Gradients in mineral concentration were observed in this secondary mineralization process, and incremental growth lines reminiscent of other normal and pathological calcifying tissues [22-27,33] were also visible. Although the exact time scale in DM is not always known, we observed in this study that the small island morphology is dominant in deposits that resided for relatively short times and continuous mineral morphology in deposits of very long duration.

Biological mineralization involves the replacement of tissue water space with mineral. The less 'protein' present per unit volume and the more water in the 'matrix', the more the matrix can be mineralized. Thus dental enamel, which loses its protein matrix during mineralization, is the most densely calcified tissue [27,28]. Cartilage matrix contains highly hydrated protein polysaccharide complexes and comparatively little collagen compared with bone and dentin, but when mineralized, it is more densely mineralized [28]. Soft tissue cells contain about 10% to 15% protein solids, and soft tissue collagen is less densely aggregated than in bone and dentin matrix. This explains the present findings that calcified soft tissue areas are more or less intermediate in position in terms of the density of calcification between bone, dentin and enamel, as well as in tissue stiffness. There was no observable difference between the modulus versus mineral concentration behavior for the two samples tested (Calc3 and Calc4, Figure 8d). This implies that the mineral in these deposits has a similar ultrastructural arrangement. Mineral concentration is important in explaining stiffness of calcified tissues, and we confirm that there is a linear correlation (R^2 ^= 0.58) between mineralization and modulus in the densely mineralized regions (Figure 8d). The more water that is displaced and the more robust the ultimate mineral particles, the stiffer a tissue becomes. However, the continuity and/or contiguity of the individual mineral particles also directly influence this parameter [39]. Thus an explanation for variations in stiffness at the same mineral concentration includes variations in mineral crystal contact [28]. The steep rise in stiffness observed at a mineral density greater than 2.5 g/ml suggests a percolation threshold; that is, sharply increased continuity of the stiff phase by contact between mineral particles. The growth and impingement of adjacent crystals within dense deposits could produce such a change in stiffness and would be associated with the more well-defined X-ray diffraction patterns observed.

There are similarities and differences between the composition of DM deposits and other forms of pathological calcification. Although the mineral in the DM deposits is composed of homogenous and highly crystallized carbonate apatite (FTIR-RM, XRD, SEM/SDD-EDS) containing Na and Mg and traces of Cl, Al and S (SEM/SDD-EDS) with Ca/P ratios of 1.63 to 1.69, explanted bioprosthetic calcified heart valves were composed of carbonate apatites containing Mg (0.06 to 0.36 wt%) with Ca/P ratios ranging from 1.34 to 2.12 [40], and with Ca/P ratios of 1.33 to 2.01 for bioprosthetic valves and of 1.62 to 2.13 for natural valves in another study [41]. SEM-EDS and XRD in this report [41] revealed that the mineral of these calcified valves was a mixture of dicalcium phosphate dihydrate, octacalcium phosphate and carbonate apatite. Carbonate apatite, β-tricalcium phosphate and octacalcium phosphate-like phase were found in different locations in the same dental calculus specimens with FTIR-RM and micro XRD [42]. Only traces of proteins were found in these specimens. Carbonate apatite was reported in another study of dental calculus as a separated phase with collagen at the calculus-cementum interface and with lower concentrations of organic phase attributed to microorganisms away from the interface [43]. Carbonate apatite was found occasionally in urinary stones as a mixture with calcium oxalate [44,45]. The concentrations of the main chemical elements in pulp stones appear to be similar to that of the DM deposits that we determined: average Ca/P molar ratios of 1.69 were reported for 10 samples of pulp stones along with 0.51 wt% Mg and 0.75 wt% Na [46]. But, although we could not detect F (the SEM/SDD-EDS detection limit for F is 0.05 wt%), the pulp stones in the above study contained 0.88 ± 0.05 wt% F and K, Cl, Mn, Zn and Fe were present at trace concentrations. Al and S (found as trace elements in Calc1 and Calc2) were reported also in soft plaque and calcified plaque deposits from human coronary arteries [47]. S was detected also in brain calcinosis along with Na, Mg and K [48]. Osteopontin, which has previously been detected in JDM deposits [8], was found in several other forms of pathological calcification: human pulp stones [49], urinary stones [50], human atherosclerotic lesions [51], and dental calculus [52]. Note that although dental calculus, pulp stones and urinary stones contain osteopontin (a known bone formation protein), they are very different from bone as well. Type I collagen was detected in the whole area of a pulp stone, while higher magnification reveled stronger staining along the growth lines of the stone [49]. Comparison of the presence of collagen in other forms of pathological calcification is more complicated. It is reported with cardiovascular deposits that were pulverized and/or powdered, but it could originate from the surrounding tissue or the heart valves tissue [40,41]. With no detailed position resolved study available, we cannot compare it with our results of the absence of collagen within the central mineralized section of the DM specimens.

Blood is supersaturated with respect to the concentrations of calcium and phosphate ions necessary to spontaneously precipitate apatite [53]. The physiological calcification inhibitors that are normally present might be missing or negated by other factors in the case of the pathological soft tissue mineralization associated with DM. Spontaneous vascular calcification, as a result of loss of mineralization inhibitors such as pyrophosphate and matrix gamma-carboxyglutamic acid (GLA) protein that are expressed normally in human blood vessels, was observed by Rutsch and colleagues [54], and spontaneous calcification of arteries and cartilage occurred in mice lacking the matrix GLA protein [55]. A recent *in vitro *study [56] showed that nanocrystals of carbonate apatite with composition and morphology analogous to atherosclerotic plaque formed *in vivo*, precipitated from human serum-like solutions that did not contain inhibitors, supporting the mechanism of spontaneous precipitation of carbonate apatite in the absence of physiological inhibitors. The waves of mineralization that we observed in our qBSE, FTIR-RM and SEM/SDD-EDS maps, and the presence of only one inorganic compound (carbonate apatite containing Na and Mg and traces of Cl, S and Al) with small amounts of proteins, might support the mechanism of spontaneous precipitation in DM calcification as well. We did not determine the nature of other inhibitors and/or nucleators present in the deposits in this study, but we might learn more in future studies.

Our new findings might aid in designing better therapies for the dystrophic calcification associated with DM. Because the findings that the crystallinity of the apatite in the DM deposits is higher than that of bone (more crystallized apatite is less soluble than poorly crystallized apatite) and its Ca/P ratios are close to stoichiometric hydroxyapatite, the presence of Mg in the deposits, the significantly smaller amount of collagen than in bone, and the possibility that the calcium and phosphate, normally present in affected tissues, may have precipitated as carbonate apatite due to local loss of mineralization inhibitors, different therapeutic agents than those that are currently used for DM may be needed to prevent, slow the progression of and/or dissolve these calcified deposits. One potential class of agents that may be of interest therapeutically are the bisphosphonates (derivatives of the biological inhibitor pyrophosphate), which inhibit the conversion of calcium phosphate into crystalline hydroxyapatite and the nucleation of hydroxyapatite [57]. This is supported by anecdotal cases of improvement in DM calcification following administration of bisphosphonate therapy [58,59]. In addition, supplemental Mg may help prevent and/or retard the progression of the calcinosis. Mg has been used to treat several other kinds of soft tissue calcifications, including myositis ossificans traumatica, calcific bursitis, traumatic osteoarthropathy, and others. Local application of magnesium sulfate into the lesion and peroral administration of magnesium lactate resulted in disappearance or reduction in size of the lesions [60]. Previous approaches have been trying to treat underlying inflammation, and we suggest using combination of bisphosphonates and other mineralization inhibitors such as Mg at the phase of rapid deposition, presumably at the peak of these waves of mineralization that we saw in our qBSE, FTIR-RM and SEM/DDE-EDS maps.

## Conclusions

The information that was obtained from the complementary FTIR-RM, SEM/SDD-EDS, XRD, μCT, qBSE imaging, nanoindentation and PLM methods increase our understanding of the dystrophic mineralization process in DM. The following observations imply that the formation mechanism of these dystrophic calcification deposits differs from that of bone: the deposits contain much less organic material than bone (FTIR-RM and qBSE); they contain Mg and Na (SEM/SDD-EDS); they contain very little collagen (PLM); the apatite is much more crystallized than bone (XRD) and closer to that in enamel; the Ca/P molar ratios are close to stoichiometric hydroxyapatite; the mineral density of the deposits (qBSE) and their stiffness (nanoindentation) are higher than bone. The formation of only one inorganic phase (carbonate apatite containing Mg and Na, by FTIR-RM and SEM/SDD-EDS) implies that the mechanism of formation also differs from those of some other pathological calcifications (cardiovascular deposits, urinary stones and dental calculus) that usually contain a mixture of inorganic compounds.

The mineral deposited first as scattered grains surrounded by tissue followed by a wave of mineralization that incorporated these particles to larger calcified bodies (FTIR-RM, qBSE, SEM/SDD-EDS, μCT).

Calcinosis masses that resided for shorter times were composed of islands of mineralization, whereas deposits with very long durations were solidly mineralized.

## Abbreviations

μCT: X-ray micro-computed tomography; 2D: two-dimensional; 3D: three-dimensional; BV: blood vessels; Calc: calcified mass; DM: adult dermatomyositis; FTIR-RM: Fourier Transform Infrared microspectroscopy in reflectance mode; FWHM: full width at one half the maximum height; H&E: hematoxylin and eosin; HP: hematopoietic marrow; IL: interleukin; JDM: juvenile dermatomyositis; JPM: juvenile polymyositis; MD: mineral density; PLM: polarized light microscopy; PMMA: poly-methyl methacrylate; PO_4_: phosphate; qBSE: quantitative backscattered electron imaging; SD: standard deviation; SEM/SDD-EDS: Silicon drift detector energy dispersive X-ray spectrometry (SEM/SDD-EDS); TNF: tumor necrosis factor; XRD: X-ray diffraction.

## Competing interests

The authors declare that they have no competing interests.

## Authors' contributions

NE participated in the design of the study, carried out the FTIR-RM and XRD studies, analyzed and interpreted the data, and drafted the manuscript. AB participated in the design of the study, carried out the qBSE studies and helped in the qBSE data analyses and morphological interpretation and in the manuscript preparation. PGTH analyzed and interpreted the qBSE data and helped in the manuscript preparation. AJB carried out the nanoindentation studies, analyzed and interpreted the data and helped in the manuscript preparation. JS carried out the μCT studies, analyzed and interpreted the data and helped in the manuscript preparation. DEN carried out the SEM/SDD-EDS studies, analyzed and interpreted the data and helped in the manuscript preparation. FWM participated in the study design and helped in the data interpretation and manuscript preparation. PGR participated in the study design, carried out the PLM studies, and helped in data analysis and interpretation and in manuscript preparation. LGR participated in the study design, coordinated the contribution of the calcified deposits from the patients' physicians, helped in data analysis and interpretation and assisted in drafting the manuscript. All authors read and approved the final manuscript.
